# Generational trends in education and marriage norms in rural India: evidence from the Pune Maternal Nutrition Study

**DOI:** 10.3389/frph.2024.1329806

**Published:** 2025-01-20

**Authors:** Akanksha A. Marphatia, Jonathan C. K. Wells, Alice M. Reid, Marios Poullas, Aboli Bhalerao, Pallavi Yajnik, Chittaranjan S. Yajnik

**Affiliations:** ^1^ UCL Great Ormond Street Institute of Child Health, Population, Policy & Practice Department, London, United Kingdom; ^2^ Department of Geography, University of Cambridge, Cambridge, United Kingdom; ^3^ Diabetes Unit, King Edwards Memorial Hospital Research Centre, Pune, India

**Keywords:** education and marriage norms, aspirations, adolescents, generational trends, educational attainment, marriage age, rural India

## Abstract

**Introduction:**

Globally in 2024, 1 in 5 women aged 20–24 years worldwide had been married before the age of 18 years. One reason for this persistent prevalence of underage marriage may be the slow change in social norms relating to education levels and women's marriage age. However, we know little about how norms change, and whether they vary by socio-demographic characteristics. We aimed to investigate changes in social norms across generations in rural Maharashtra, India.

**Methods:**

To understand the status quo, we identified education levels and marriage ages typical of contemporary young adults in rural Maharashtra using the National Family Health Survey. To see if norms have shifted across generations, we analysed data on education and marriage age in 659 parent-adolescent dyads from the Pune Maternal Nutrition Study (PMNS) in rural Maharashtra. To ascertain if norms might shift in the future, we investigated adolescents' aspirations for their future hypothetical children's education and marriage, and classified adolescents as wanting (a) their children to decide themselves, (b) more education and later marriage age, or (c) the status quo. We assessed whether these aspirations differed by socio-demographic characteristics.

**Results:**

Compared to the status quo and PMNS adults, PMNS adolescents had substantially more education, and girls were marrying slightly later. About 70% of the adolescents wanted their children to themselves decide their schooling. The remainder of both sexes wanted their children to have the same education as them (15 years). Only 10% of adolescent girls and 14% of boys wanted their child to decide their own marriage age. Most adolescents wanted a later marriage age for their children than their own experience. Lower educated and early married girls aspired for greater education for their children. More educated boys aspired for later marriage for their children.

**Discussion:**

Education norms have changed by a larger magnitude than marriage age norms. Adolescents are already attaining their education aspirations, but aspire for later marriage of their children, more so for their hypothetical sons than daughters. Since senior household members remain influential in marriage decisions, it may take time before adolescents' aspirations for their children become a new norm.

## 1 Introduction

### 1.1 Background and rationale

Globally in 2024, 1 in 5 women aged 20–24 years had married before the age of 18 years (1). One in three of the world's early married women lives in India (2). Early marriage is the target of a range of policies, due to its adverse associations with maternal and child outcomes. For example, early married women generally have less education, reside in rural areas, and marry into poor households (2), and have poorer nutritional status and a higher risk of preterm birth and poor growth in their offspring (3–12). In India, efforts to delay girls' marriage have contributed to a decrease in marriages <18 years from 47% in 2006 to 23% in 2019–2021 (13, 14). These efforts include legislation mandating 18 years and 21 years as the minimum marriage age for girls and boys respectively (15). In the past decade, the proportion of girls' completing higher secondary education, the key target of efforts to delay girls' marriage (6, 16), has increased from 34% to 48% (17). However, this increased education has yet to lead to similar decreases in early marriage (18).

Current progress in delaying the age at which girls marry would need to be substantially accelerated to meet the Sustainable Development Goal of eliminating early marriage by 2030 (19). Maharashtra state, where our study is based, has the fourth highest proportion (22%) of early married women in India (2). Over the past decade, the average annual rate of reduction in the percentage of women in Maharashtra married early was 3.9%, whereas eliminating the practice by 2030 would require an annual reduction rate of 34.3% (20).

One possible reason for the slow progress in delaying girls' marriage may be that social norms regulating education and marriage are slow to change (21, 22). A major challenge in studying norms is that they are represented in different ways at different levels of society, as defined below. For example, individuals may express values and preferences, through appealing to cultural criteria of custom and convention, whereas national institutions may try to enforce norms using legal requirements. The product of social norms is also visible in the statistical frequency of outcomes, reflecting the consequence of numerous individual decisions. For education, for example, households may prefer a certain number of years of schooling for their child; the state may mandate a certain minimal level of education; and surveys can document the level of education most commonly attained in the population, as well as its variability. All of these aspects of norms may change over time.

### 1.2 Defining norms

Socio-cultural norms reflect what people believe to be the ideal behaviour in their society, though these norms often differ for the two sexes (23, 24). Socio-cultural norms are powerful because they are self-sanctioning: any individual who deviates from them may be socially isolated or ostracised (21). In the Indian context, for example, non-adherence to marriage norms may have repercussions for marrying other children in the family (25). Socio-cultural norms tend to be instilled in individuals at a young age by older influential members of the household, and are therefore resistant to change (26, 27). Any changes in these norms are likely to be gradual, with an increasing number of individuals deviating from or rejecting the norm eventually leading to a collective shift (22).

Legal norms, such as the minimum marriage age, are formal declarations institutionalised by the state, though not always enforced (22). These legally instituted norms aim to construct and enforce socio-cultural norms, for example by mandating a minimum schooling level or marriage age. In India, the minimum legal age of marriage for girls is 18 years and for boys, 21 years (15). This difference in the minimum marriage age of the two sexes not only permits, but essentially legalises, societal gender differences in the timing of this key life event, which often marks the social transition to adulthood (28).

Overall, evidence of legislation driving decreases in early marriage remains weak (29), despite increasing examples of punitive measures being implemented (30, 31). Instead, the data described in the background section indicate that, notwithstanding legal marriage norms, socio-cultural norms in India continue to drive decisions that, for a proportion of women, result in early marriage.

Rather than changing on the basis of legislation, socio-cultural norms could also change because of shifts in cultural values regarding what is acceptable and desirable. As education levels rise, for example, today's adolescents may increasingly aspire for a different experience for their own offspring in the future.

### 1.3 Aspirations

Aspirations differ from norms in expressing an individual's hopes, dreams, ambitions or goals of what they would like to become or achieve (32–34). They tend to be future-oriented and look towards attaining a particular trajectory, and can be driven by conscious and unconscious motivations (32). Individuals may set their aspirations in relation to what they know that they can achieve, to fit existing behaviours or policies, or strive more ambitiously for things they are not sure of realizing (32). Aspirations may be shaped and constrained by different factors (35), and depending on the context, they can either be socially constructed or transformative. If such aspirations were to be commonly realized, they would emerge as new socio-cultural norms, reflected in new outcomes in the population.

The aspirations of adolescents are important to consider because they may have little opportunity to contribute to formal discussions about norms and decisions around their own education and marriage, even as they pass through the very life stage when these decisions are made. Moreover, their aspirations are likely to reflect the agency available to them, and the values held by others in their immediate social environment (36). Factors such as gender and societal status may shape the freedom of individuals to develop aspirations, as well as their capacity to realise them (32).

### 1.4 Challenges to studying aspirations and norms

Both norms and aspirations are inherently difficult to research. Directly asking individuals about their aspirations may cause distress if they are asked about opportunities that were already denied to them. In Ghana, for example, girls who perceived that they had been married too young reported negative emotions (37). To address this, studies have either not asked about self-aspirations (35), or depersonalized aspirations by asking about the “ideal” age at marriage in the third person, or asked adolescents to “imagine” aspirations without considering any constraints (25, 35, 38–40). These challenges mean that we still know little about adolescents' own aspirations, how they may change, and whether they differ from prevailing social norms. Understanding potential gender differences in both norms and aspirations is also critical, because many decisions over women's lives are taken by men.

The consequence of the expression of socio-cultural norms in the past is evident in a population's characteristics (e.g., typical marriage age), reflecting the outcome of common decisions. Examining trends in these outcomes across generations can indicate how norms and behaviours have changed over time. Many studies have examined generational changes in educational attainment and marriage age using nationally representative data (6, 41–43). In rural Nepal, for example, generational trends in the transition to womanhood (age at marriage, co-residence and first childbearing) amongst women residing in the same household were evaluated (44).

Here, we bring these two approaches together to improve understanding of both how socio-cultural norms have been changing in rural Maharashtra, and how they might change further in the future, if adolescents' aspirations were to be realised. We also seek to understand what characteristics of adolescents own life trajectories are associated with their desire to shift the status quo.

### 1.5 Objectives of study

This study aims to contribute new insight into whether norms on education and marriage age are changing over time in rural Maharashtra. To explore whether norms are changing over time, we looked at habitual practice, or the activation of normative decisions across two generations. To understand whether adolescents aspired to change these norms further in the next generation, we asked what outcomes they wanted for their *future hypothetical* children, who we refer to as *hypothetical sons* and *daughters* throughout the rest of this paper. We also wanted to understand whether the likelihood of adolescents trying to shift social norms depends on their gender and their own education and marital status.

We tested the following three research questions:

(1) Have norms changed over time, as reflected by numerical values (duration of education, girls' marriage age) and prevalences (school dropout, early marriage)?
(2) Do adolescents aspire for further changes in norms for their future hypothetical children?
(3) Are more educated and unmarried adolescents more likely to desire greater changes in these norms?

## 2 Methods

Our analysis used data from two sources. To investigate socio-cultural norms at the individual level, we analysed data from the Pune Maternal Nutrition Study (PMNS), a prospective longitudinal birth cohort based in six villages of Pune district in Maharashtra state, India, as described below. To understand the broader norms that may shape decisions in the PMNS cohort, we also analysed data for rural Pune and Maharashtra from the fourth Indian National Family Health Survey (NFHS), collected in 2013–2014 (45).

### 2.1 Study design and setting

The PMNS aims to understand how maternal nutrition influences fetal growth and offspring susceptibility to diabetes and cardiovascular disease (46–49). The study recruited mothers between 1994 and 1996, prior to conception of the cohort child, and then followed the children from birth onwards (50). Cohort children were followed-up at the ages of 2, 6, 12, 18 and 24 years.

In 1993, around the inception of our study, 54% of women aged 20–24 years had married <18 years of age in Maharashtra, and the median age at first marriage of these women was 17.5 years (51). About 44% of women (aged 6+ years) residing in Maharashtra were illiterate (51). The society that encompasses the PMNS is patriarchal, with inheritance passed down the male line. Girls typically end their schooling and move on marriage into their husbands home in which his parents also co-reside (52). Women generally have low decision-making and autonomy in households, work long hours on both unpaid care work in the home (cooking, cleaning childcare, etc.) and as farm labourers, and tend to eat last and least (53, 54). Most households were subsistence farmers and owned small landholdings (53).

Local village leaders, the Indian Health Ministry Steering Committee and the Ethical Committee at the King Edward Memorial Hospital Research Centre (KEMHRC) (VSP/Dir.Off/EC/2166) granted ethical permission to conduct the PMNS. Additional approval for the collection of adolescent's educational and marriage trajectories, and aspirations at the 18-year follow-up was granted by the Indian Health Ministry Steering Committee, KEMHRC and the Research Ethics Committee in the Department of Geography at the University of Cambridge. At the 18-year follow-up, adolescents at or above majority age of 18 years gave written consent to participate in the study. For those who were below the majority age, a parent/guardian gave written consent for the adolescents' participation in the study. These younger adolescents also gave their own written assent (or agreement) to participate in the study, and upon turning 18 years, gave additional written consent.

### 2.2 Variables

Data on household characteristics come from the time of recruitment. An oral questionnaire was administered to married non-pregnant women upon recruitment into the PMNS. Household characteristics were collected at baseline and included women's age at marriage (years) and both their own and their husband's educational attainment (years of schooling completed). Caste and household wealth were measured according to the widely used and standardized Pareek and Trivedi 'Socio-economic Status Scale' for rural India (55, 56).

We use data on adolescents' age (years), educational attainment, and marriage age (years) from the 18-year and 24-year follow-up. For those still in school, we asked whether they aspired to complete the 12th standard. Data were collected on the aspirations that adolescents held for their future children's education and marriage age. We adopted this approach because during the pilot study, two married girls were visibly distressed by being asked questions that reminded them that their own life aspirations had been unattained due to their early marriage.

Adolescents could provide 1 of 3 possible responses on the aspirations they held for their future hypothetical children:

• They wanted their hypothetical children to choose for themselves how much schooling to complete and when to marry
• They specified numerical values for how many years of schooling they wanted their hypothetical children to complete, and the age at which they wanted them to marry
• They did not know

We also asked adolescents to give a free-text response for why they wanted their future daughters and sons to marry at different ages.

### 2.3 Data coding

To describe our sample, we coded parental education according to the Indian education system: none (0 years completed), Primary (1–5 years), or Secondary or higher (≥9 years). Caste affiliation was coded as per the Pareek and Trivedi Socio-Economic Status Scale as low (tribal or scheduled), mid (artisan or agrarian) or high (prestige or dominant) (55, 56). A composite score of household wealth was derived from weighted scores of individual assets using Principal Component Analysis (57).

Depending on the analysis, we described the educational attainment and marriage age of adolescents as a continuous value, in years, or as a categorical variable: stopped studying by 18 years (yes vs. no) and unmarried by 19 years vs. married early, <19 years. Although the minimum marriage age of women in India is 18 years (15), as only a few of the adolescent girls in our sample had married by this age, we used 19 years as the cut-off. This approach has been used in other studies (58, 59).

The reasons that adolescents gave for wanting a different age at marriage for their hypothetical daughters and sons were grouped into 6 themes which emerged from the data: (a) boys should marry later than girls because they have to be financially settled before marrying; (b) girls marry earlier because they are mentally and physically mature at an earlier age than boys; (c) there would be problems if girls married later; (d) girls should marry immediately after completing their secondary education; (e) to respect traditional social norms; or (f) girls should marry right after the minimum legal marriage age of 18 years.

### 2.4 Coding norm outcomes

To categorise norm outcomes, we used a modelling exercise to propose norm values and to test our data against them. Our approach is similar to the method used for defining obesity (e.g., BMI >30 kg/m^2^) (60, 61), which allows (a) the analysis of BMI as a continuous variable, (b) the analysis of the prevalence of obesity, and (c) the identification of predictors of variability in both BMI or obesity prevalence. We used a similar approach for categorising norms and assessing their change over time.

First, we quantified the most common outcomes in rural Maharashtra using the fourth Indian National Family Health Survey (NFHS), collected around the same time of the 18-year follow-up in our study, in 2013–2014 (45). We restricted the sample of women and men to 21–24 years of age as this is the age group where the 18-year-olds in our study could see current norms being practiced. To obtain a sufficient sample of men, we used all rural districts in Maharashtra because there were only 8 men aged 21–24 years in the rural Pune NFHS sample with marriage age data. This yielded a sample of 101 men aged 21–24 years with data on marriage age and 97 men with data on education. For women, there was a sufficient sample of women aged 21–24 years residing in rural Pune district (*n* = 64), where our study was conducted. We did not expand this sample to rural Maharashtra because the modal years of women's education and marriage age were higher than rural Pune.

**Figure 1:**
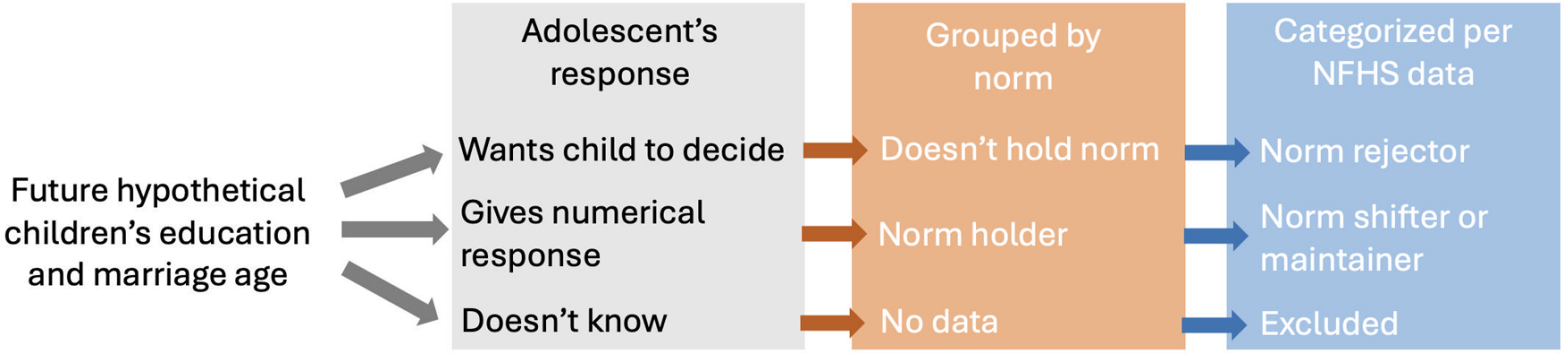
Decision-tree describing coding of norm outcomes. This decision-tree was used to categorise norms outcomes. When asked about future hypothetical children's education and marriage age, adolescents could provide one of three responses (grey box). We grouped these responses into two types of norms (orange box), and then categorised our sample into three groups using data from the Indian National Family Health Survey (NFHS) (blue box).

In the NFHS-4 survey (45), the mode (most common) years of schooling completed for women was 5 and for men, 4 years. The mode age at marriage of women was 17 years and of men 21 years. Using these data, for each of education and marriage age, we then assigned the adolescents in our study into one of three categories, as illustrated in Figure 1:

- ‘Norm rejectors’ did not want to specify numerical values for the outcomes, instead they wanted their hypothetical children to make the relevant decision
- ‘Norm shifters’ specified numerical values for the outcomes, and wanted their hypothetical children to have greater education and marry later than the population norm. We specified the threshold for 'shifting' as desiring ≥2 years above the NFHS mode (to allow for error/noise), i.e., they desired ≥7 years of education for future daughters and ≥6 years for future sons, and marriage age of ≥19 years for daughters and ≥23 years for sons.
- ‘Norm maintainers’ specified numerical values for the outcomes, and wanted similar education and marriage age for their hypothetical children as the population norms. This group had values of <2 years above the NFHS mode, equivalent to ≤6 years of education for hypothetical daughters and ≤5 years for sons, and marriage age of ≤18 years for daughters and ≤22 years for sons.

We used these three categories to assess whether adolescents wanted to reject, shift or maintain current education and marriage norms for their hypothetical children.

### 2.5 Statistical methods

To describe our study population, we used the mean [standard deviation (SD)], Frequencies (F) and percentages (%). We used chi-square tests to evaluate differences in categorical outcomes.

For our first research question on generational trends, we first compared the most common number (mode) for education and marriage age in the full sample of cohort parents and adolescents, and for the subset of adolescents who gave a numerical number for what they aspired for their hypothetical children. Second, we described the proportion who had left school before the 10th standard and married <19 years across these three generations.

For our second research question, we first quantified the proportion of adolescents who rejected prevailing norms, wanted to shift them beyond the status quo, and wanted to maintain the status quo, by adolescent sex and by the sex of their hypothetical children. Second, we compared mean values in how much education adolescents wanted their children to complete and the age at which they wanted them to marry, and used paired *t*-tests with 95% Confidence Interval (CI) to evaluate differences in by adolescent sex and their hypothetical children's sex. We used independent samples *t*-tests (with 95% CI) to assess differences in these values between adolescents who wanted to shift vs. maintain norms. Third, we explored why adolescents wanted their hypothetical daughters and sons to marry at different ages.

For our third research question, we first investigated whether the norms held by adolescents regarding their children's education and marriage differed by the adolescent's own educational status, sex, and the sex of their hypothetical children. Second, we investigated differences in hypothetical children's norms differed by the adolescent girls' own marital status.

## 3 Results

### 3.1 Participants

The study flowchart in Supplementary Figure S1 describes the number of mothers and adolescents available for our analysis at different time-points. Data on maternal and household characteristics were collected at baseline (shown in the grey box), and varied in sample size from 629 to 644. We excluded 7 mothers with unverifiable marriage age of <10 years when describing maternal marriage age. Data on adolescents are shown in orange boxes, and include 659 adolescents at the age of 18 years and 642 adolescents at 24 years.

### 3.2 Descriptive data

Table 1 describes household and adolescent characteristics. Sixty-five percent of mothers had married early (<19 years), 22% were uneducated, 51% had completed primary education, and 27% had completed secondary or higher education. Nine percent of fathers were uneducated, 36% had completed primary education and 55% had completed secondary or higher education. Eight percent of households were from lower caste affiliation, 23% and 69% from mid and high affiliation respectively. Thirty percent were from low SES and 35% from mid and low categories respectively. The average years of schooling completed by adolescents by the age of 19 years was 12 years (SD 1.6), and 12% had not completed lower-secondary school (10th standard). Eleven percent of girls had married <19 years, at mean age 18.7 years (SD 1.4). Only 1 boy had married <19 years, at 18.4 years. Supplementary Table S1 shows that there were no differences by adolescent sex in household or adolescent traits.

**Table 1:** Description of study sample (*n* = 659).

Household traits	F	%
Maternal marriage age (years) (missing *n* = 30)		
<19 years	410	65
≥19 years	219	35
Maternal education (years) (missing *n* = 33)		
None	136	22
Primary (1–8 years)	318	51
Secondary or higher (≥9 years)	172	27
Paternal education (years) (missing *n* = 33)		
None	59	9
Primary (1–8 years)	227	36
Secondary or higher (≥9 years)	340	55
Caste affiliation (missing *n* = 15)		
Low (tribal, scheduled)	54	8
Mid (artisan, agrarian)	149	23
High (prestige, dominant)	441	69
Socio-economic Status (missing *n* = 16)		
Low	197	30
Mid	225	35
High	225	35
Adolescent's traits	Mean	SD
Education (years) at 18 years (missing *n* = 130)	12.0	1.6
Age at marriage (years) at 19 years^a^	18.7	1.4
	F	%
Did not complete 10th standard	81	12
Married <19 years	72	11

*n,* number; F, frequency; %, percentage; SD, standard deviation.

^a^ Girls *n* = 133, boys *n* = 1.

### 3.3 Research question 1

**Figure 2:**
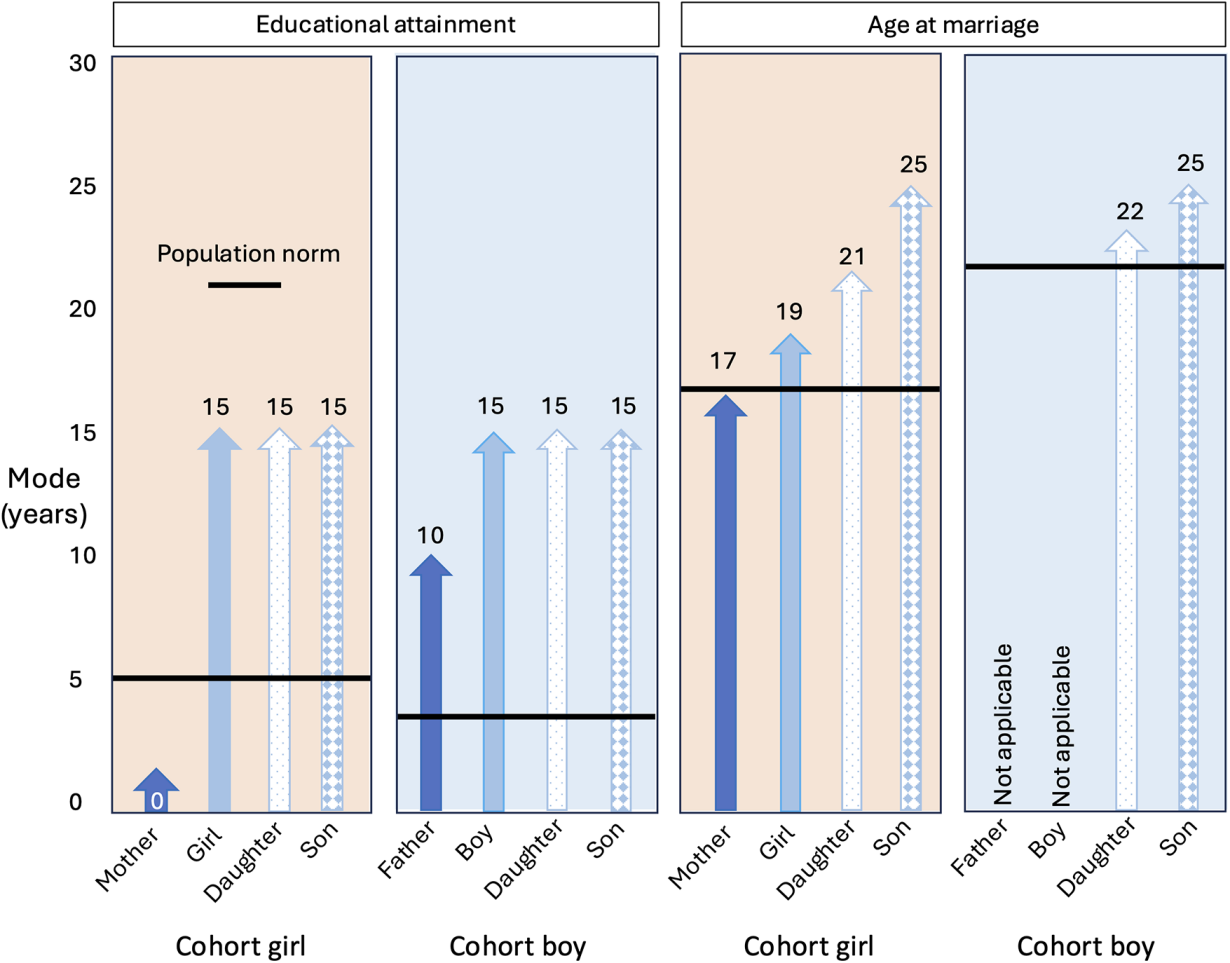
Data on cohort parent and adolescent education are for the full sample, and data on adolescent girls' marriage age are for the 90% who were married by the age of 24 years, and indicate generational changes. Data on adolescent's hypothetical children are on a subset who gave a numerical response, and indicate what adolescents aspired for their hypothetical children. Pink shaded boxes describe trends across females and blue shaded boxes trends across males. Across all boxes, cohort parents are shown in dark blue, cohort adolescents in the light blue, adolescent's hypothetical daughters in the dotted blue and sons in blue diamonds. We did not collect data on the cohort father's age at marriage. The modal marriage age for boys was 26 years, but as only 27% of boys were married, the mode is very likely to be greater in the future.

To test our first research question on generational changes in attained education and marriage age, we first contrasted the mode, or most common value for cohort parents and adolescents in the whole sample. Figure 2 shows substantial generational increase in female and male modal educational attainment, and a smaller increase in female modal marriage age.

We then compared these generational changes with adolescents' aspirations for their hypothetical children in a subset of adolescents who had given a numerical response (ie those who wanted to either shift the status quo or maintain it). Analyses of these numerical values necessarily excluded those adolescents who wanted their children to decide their own education level and marriage age (norm rejectors, Supplementary Table S2). For education, compared to norm rejectors (*n* = 429), those who provided numerical response for their children's education (*n* = 189) were more like to have dropped out of school (OR 1.9, 95% CI 1.1, 3.1) and to have been married earlier (OR 2.3, 95% CI 1.4, 4.0). However, those who specified a marriage age (*n* = 548) did not differ in their likelihood of school dropout or early marriage compared to norm rejectors (*n* = 70).

We found that both adolescent girls and boys most commonly wanted the same level of education for their hypothetical daughters and sons as the modal value of the whole cohort. Both adolescent girls and boys most commonly wanted their hypothetical children to marry later than the cohort modal age. Both adolescent girls and boys wanted daughters to marry before sons, though adolescent boys wanted a slightly smaller age-gap (3 years vs. 4 years) compared to girls.

**Figure 3:**
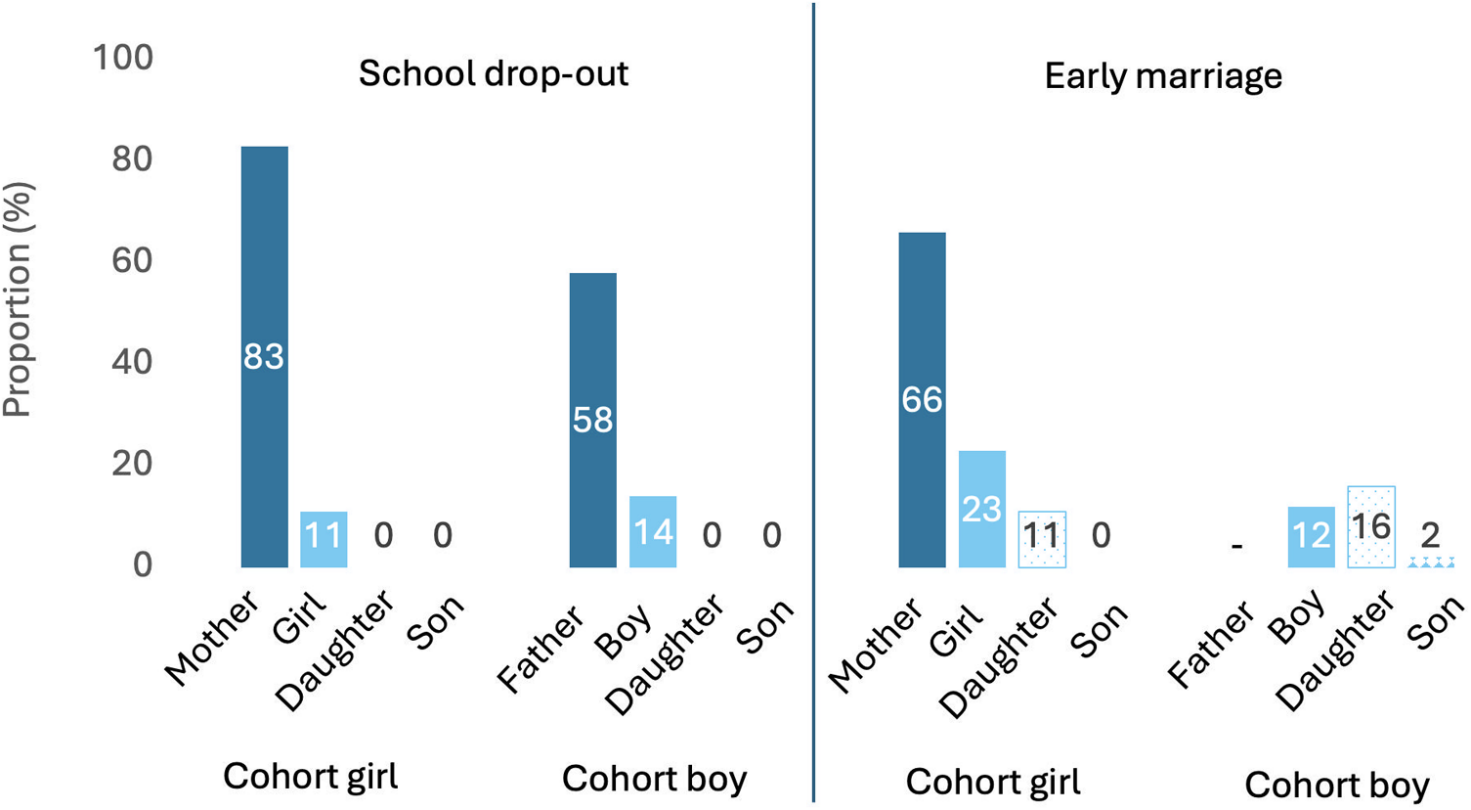
Generational trends in school drop-out and early marriage. The panel to the left shows generational trends in school drop-out, and the panel on the right, early marriage. Data on adolescents is from the 24-year follow-up, with early marriage for girls defined as <19 years of age and for boys, <21 years. Across both panels, cohort parents are shown in dark blue, cohort adolescents in light blue, adolescent's hypothetical daughters in dotted blue and sons in blue diamonds. We did not collect data on the cohort father's age at marriage.

Second, we investigated whether there were generational changes in the full sample of cohort parents and cohort adolescents in the proportion dropping out of school (not completing the 10th standard) and marrying early (<19 years for females and <21 years for males). For the subset of adolescents who gave a numerical response, we then described the proportion wanting their hypothetical children to leave school and marry early. Figure 3 shows substantial generational declines in the proportions of females (from 83% to 11%) and males (from 58% to 14%) dropping out of school, and of females marrying early (from 66% to 23%). The decline in school drop-out was greater amongst females than males. Amongst females, the decline in school-drop was also greater than that in early marriage. Compared to adolescent girls, a greater proportion of adolescent boys wanted their hypothetical daughters and sons to marry early.

### 3.4 Research question 2

To extend the investigation beyond these modal values, which for education were provided only by a subset of adolescents, we next quantified in more detail what the adolescents wanted for their hypothetical children's education and marriage, and why.

Of the 659 adolescents included from the 18-year follow-up, we could not include 41 who responded that they “did not know” how much education they wanted their hypothetical children to complete and the age they wanted them to marry, thus preventing us from assigning them to any of the three norm groups. Adolescents who did not know their aspirations for their hypothetical children were more likely to have dropped out of school (OR 3.2, 95% CI 1.6, 6.7) and married early (OR 2.9, 95% CI 1.4, 6.2) compared to those who gave a response (Supplementary Table S3). This approach resulted in analysing 618 adolescents, of which 286 (46%) were girls.

**Figure 4:**
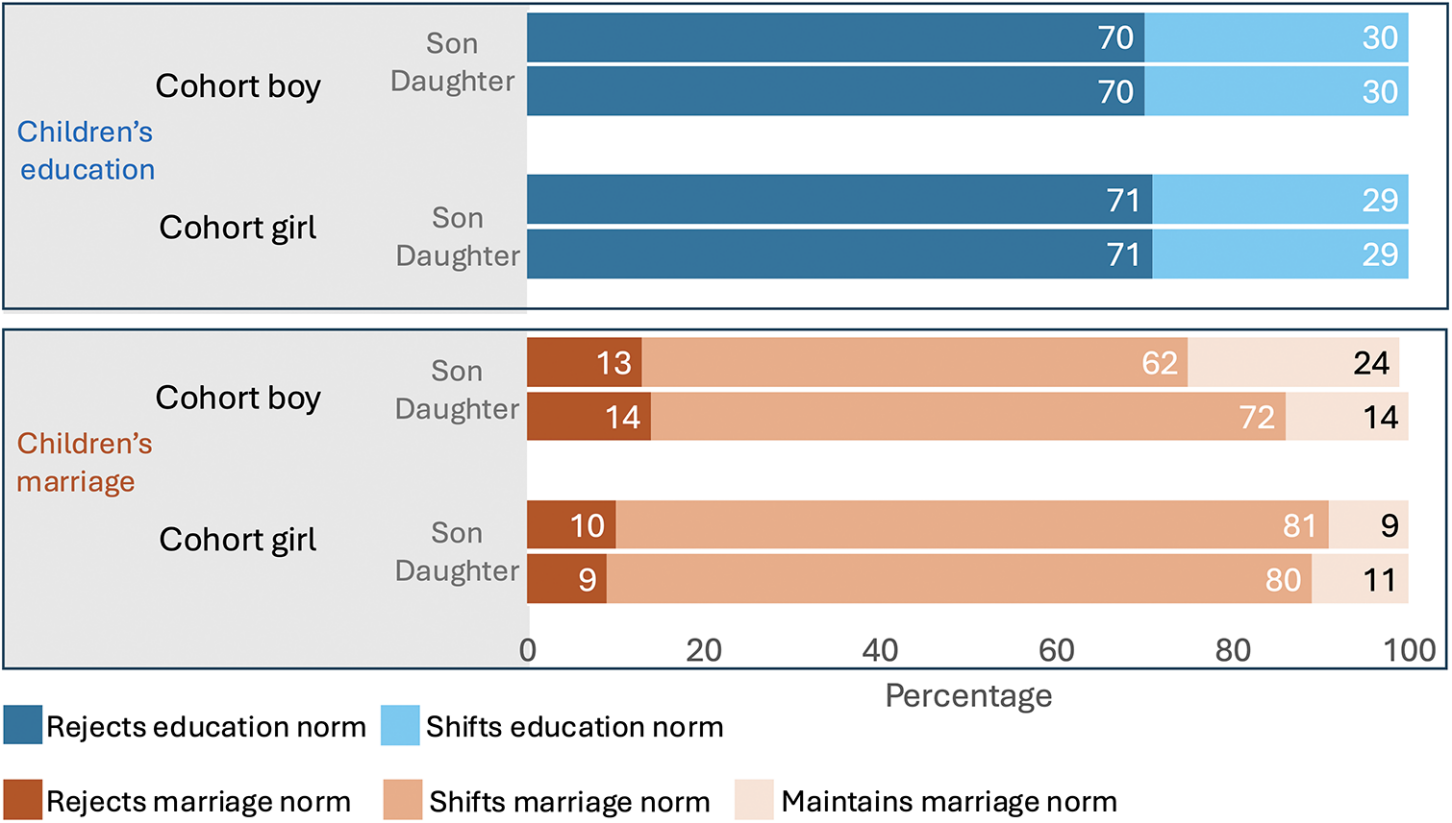
Adolescent norms for their future hypothetical children's education and marriage age by adolescent's sex. The top panel shows adolescent's norms for their hypothetical children's education and the bottom panel norms for children's marriage, by adolescent sex, and children's sex.

Figure 4 shows that amongst these adolescent boys and girls, ∼70% rejected current education norms by wanting their hypothetical children to decide for themselves how much schooling to complete, ∼30% wanted to shift education norms beyond the status quo, and none wanted to maintain the status quo. There were no differences by either adolescent sex or hypothetical children's sex in education norms.

In contrast, a minority of adolescent girls and boys (∼10%) wanted to reject marriage norms. Adolescent boys showed a greater tendency to challenge the status quo for their daughter's marriage (72%) compared to their son's (62%), and wanted to maintain marriage norms for sons (24%) more so than daughters (14%). A higher proportion of adolescent girls compared to boys wanted to shift marriage norms (∼80%), but they wanted similar outcomes for both their daughters and sons.

Second, we investigated sources of variability in these norms, by comparing the mean values of numerical norms between those who wanted to shift vs. maintain norms, and by the sex of the adolescent's hypothetical children (Table 2).

**Table 2:** Years of education and marriage age specified for future hypothetical children, stratified by whether the adolescent wanted to shift or maintain the norm, the adolescent's sex and the future child's sex (*n* = 618).

	Adolescent boys			Adolescent girls		
	Education of hypothetical children					
Daughter	*n*	Mean	SD	*n*	Mean	SD
Shifts norm – wants more than status quo	105	15.2	2.0	85	15.6	1.1
Maintains norm – accepts status quo	0	–	–	0	–	–
Rejects norm – child decides^a^	235	–	–	206	–	–
Son						
Shifts norm – wants more than status quo	102	15.7	2.1	82	15.7	0.9
Maintains norm – accepts status quo	0	–	–	0	–	–
Rejects norm – child decides^a^	235	–	–	206	–	–
	Marriage age of hypothetical children					
Daughter	*n*	Mean	SD	*n*	Mean	SD
Shifts norm – wants more than status quo	251	21.9	2.1	276	22.4	2.0
Maintains norm – accepts status quo	48	18.0	0.1	31	18.0	0
Rejects norm – child decides^a^	33	–	–	14	–	–
Son						
Shifts norm – wants more than status quo	218	25.3	1.6	247	25.9	1.9
Maintains norm – accepts status quo	81	21.4	0.8	26	21.4	0.5
Rejects norm – child decides^a^	33	–	–	13	–	–

*n*, number; SD, standard deviation; –, not applicable.

^a^ Adolescents in the “rejects norms” category did not give a numerical value for their future hypothetical children's education and marriage age.

None of the adolescents wanted to maintain education norms, hence all those analysed were norm shifters, and on average they wanted their future children to have ∼15 years of education. Paired *t*-tests showed no difference in the education desired by adolescent girls for their hypothetical daughters and sons, but on average, adolescent boys wanted their hypothetical daughters to complete −0.5 years less schooling than sons (95% CI −0.7, −0.2).

Independent samples *t*-tests showed that on average, adolescent boys and girls wanted their hypothetical daughters to marry 3.0 years (95% CI −3.1, −2.8) and 3.6 years (95% CI −3.8, −3.4) earlier than their future sons, respectively. Paired *t*-tests showed that adolescent boys who wanted to shift norms wanted their offspring to marry on average ∼4 years later than norm maintainers (daughters 95% CI 3.6, 4.2; sons 95% CI 3.7, 4.2). Similar patterns were apparent for adolescent girls, with those wanting to shift norms wanted their hypothetical children to marry on average ∼4.4 years later (daughters 95% CI 4.2, 4.7; sons 95% 4.2, 4.2) compared to norm maintainers.

Third, we explored the reasons why adolescents wanted their hypothetical daughters and sons to marry at different ages. For this analysis, we excluded 35 adolescents who wanted their daughters and sons to marry at the same age; 2 who wanted their daughters to marry later than sons; and 100 who did not provide any explanation. There were no differences in household or adolescent characteristics between those included vs. excluded from this analysis.

**Figure 5:**
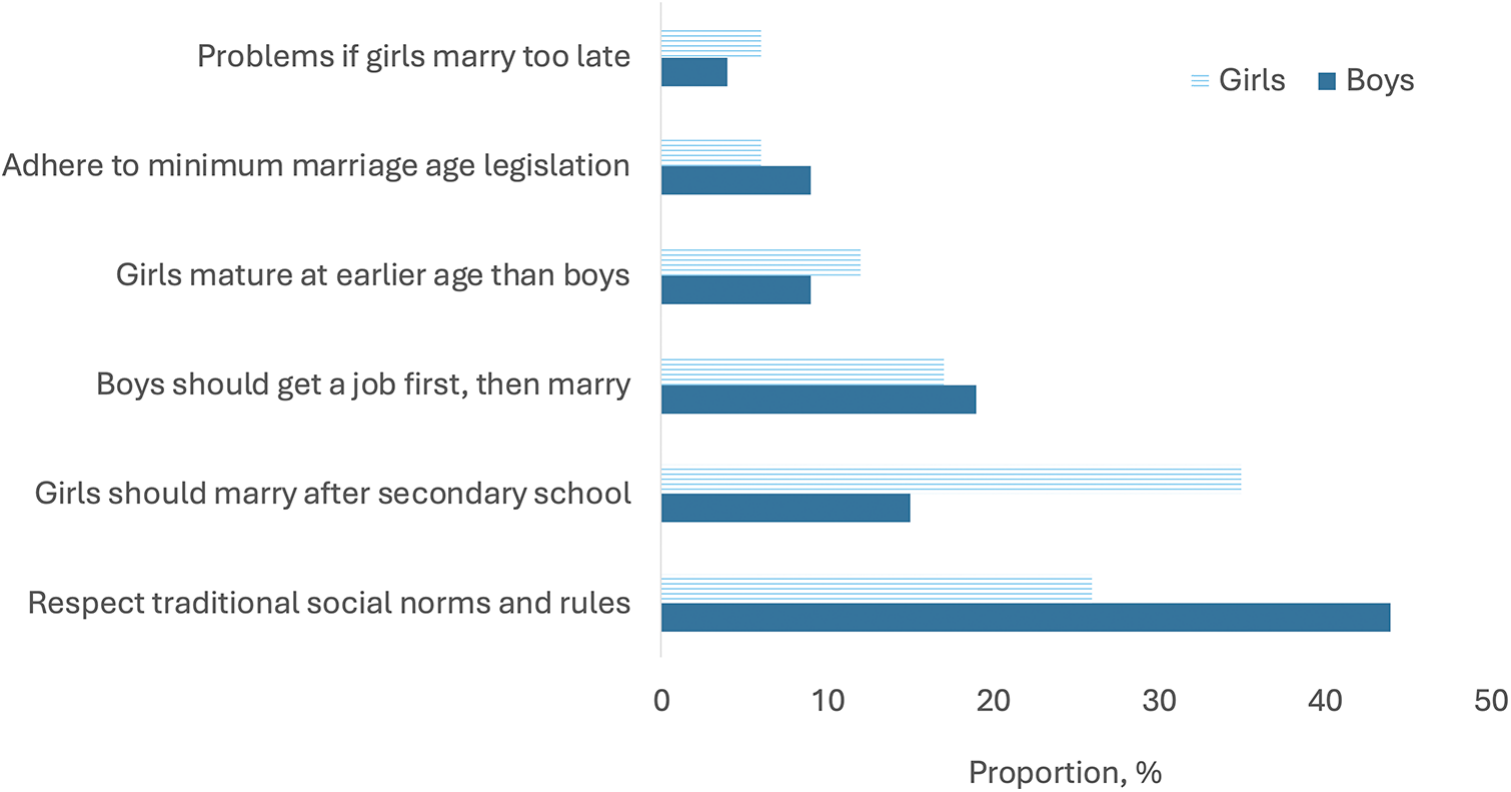
Reasons given by adolescents for wanting girls to marry earlier than boys. The reasons given by adolescents for girls marrying earlier than boys are stratified by adolescent boys (dark blue bar) and girls (lined blue bar).

Among those analysed (*n* = 435), Figure 5 shows that a greater proportion of adolescent girls compared to boys thought that girls should marry immediately after completing their secondary education, whereas boys did not face a similar pressure. Among the problems that girls were considered to encounter if delaying marrying were: unwanted teasing and sexual harassment by boys; gossiping in the community around why she was not married; trouble finding a husband, partly because their “looks” would fade with age; and difficulty conceiving children. A greater proportion of adolescent boys, compared to girls, thought that girls should marry earlier than boys out of respect for traditional norms; right after the minimum marriage age legislation for girls; and because boys should get a job and be financially settled before marrying.

### 3.5 Research question 3

**Figure 6:**
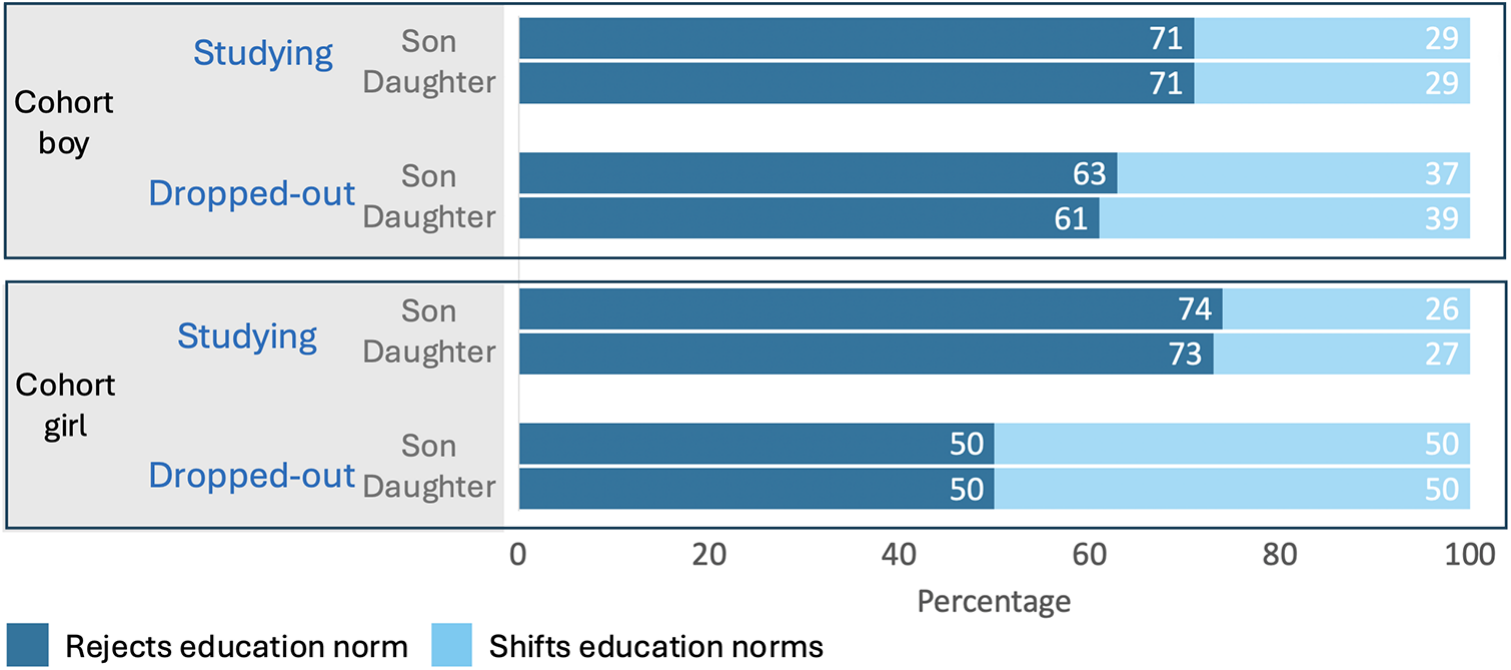
Adolescent girls’ and boy's norms for their future hypothetical children's education by adolescent's own educational status. The top panel shows adolescent boy's norms for their hypothetical children's education and the bottom panel norms held by adolescent girls, by adolescent's educational status, and children's sex.

For our third research question, we first investigated whether the norms held by adolescents regarding their children's education differed by the adolescent's own educational status. Figure 6 shows that adolescents of both sexes who had dropped out of school were less likely to reject the norm altogether, and more likely to want to shift it, for both sons and daughters. Beyond that, the results were similar for adolescent boys and girls if they were still studying, whereas boys who had dropped out were more likely to reject the norm, compared to girls who had dropped out. None of the adolescents wanted to maintain the status quo for their hypothetical children.

**Figure 7:**
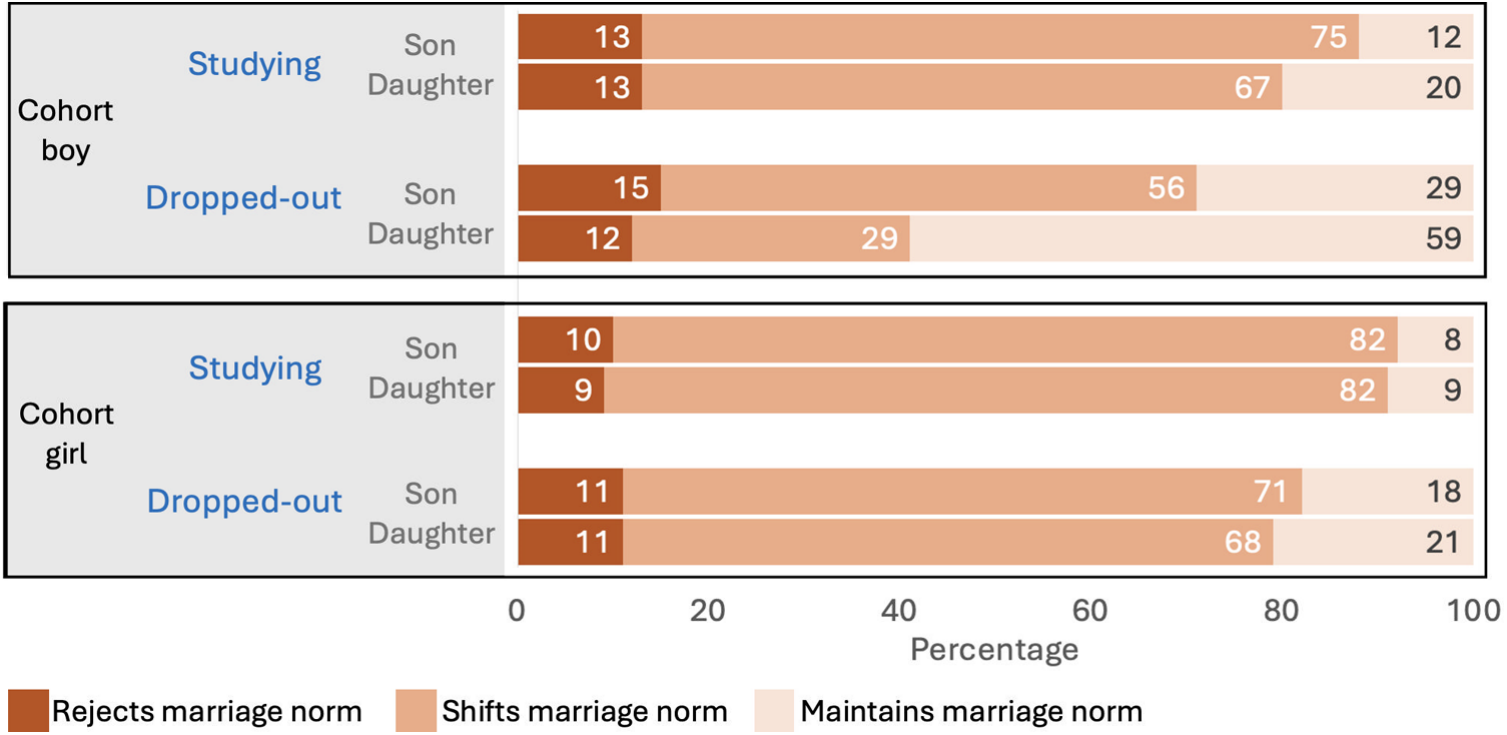
Adolescent girls’ and boy's norms for their future hypothetical children's marriage age by adolescent's own educational status. The top panel shows adolescent boy's norms for their hypothetical children's marriage and the bottom panel norms held by adolescent girls, by adolescent's educational status, and children's sex.

Second, we investigated whether the norms held by adolescents regarding their children's marriage differed by the adolescent's own educational status. Figure 7 shows that only a small minority of the adolescents wanted to reject marriage norms altogether. Rather, the majority of adolescents of both sexes wanted to shift marriage norms, but the frequency was lower among those who had dropped out of school, as in this group a higher proportion were happy with the status quo. Within this overall pattern there were two further findings. Adolescent boys who had dropped out were less likely to want to change the norm rather than accept the status quo, compared to adolescent girls. Moreover, this sex difference was substantially greater for hypothetical daughters compared to sons, with only 29% of adolescent boys of wanting to change this norm, but 68% of adolescent girls.

**Figure 8:**
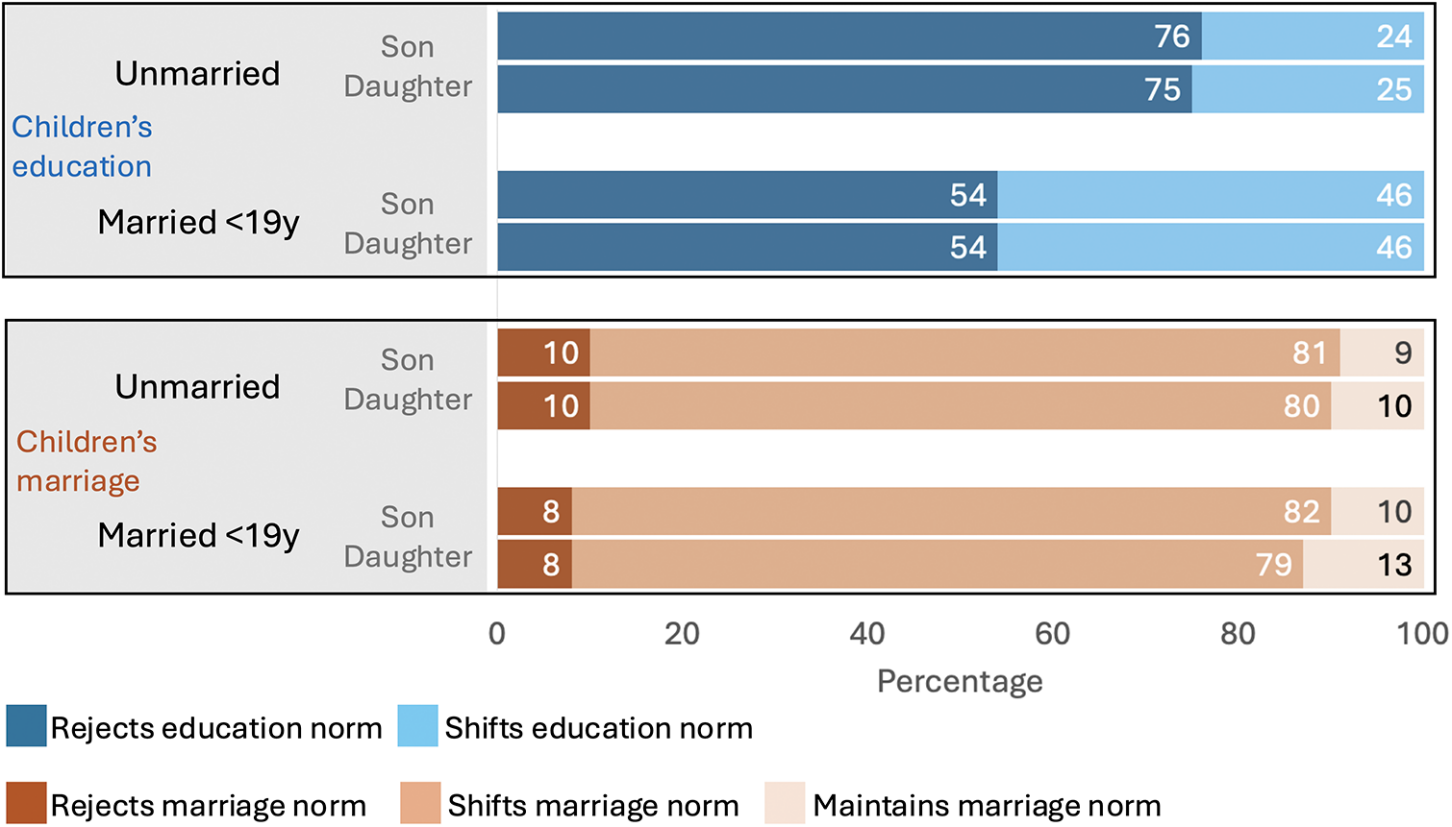
Adolescent girls' norms for their future hypothetical children's education and marriage age by adolescent girls' own marital status. The top panel shows adolescent girls' norms for their future hypothetical children's education and the bottom panel norms held for children's marriage age, by adolescent girls' own marital status.

Third, we investigated differences in education and marriage norms by the adolescents girls own marital status. Figure 8 shows that for hypothetical children's education, early married girls were less likely to reject education norms, but more likely to desire greater education than the status quo. This pattern was identical for hypothetical sons and daughters. Conversely, early married girls showed negligible differences to married girls regarding marriage norms, with both groups primarily wanting to shift the norm, and only small proportions (∼10%) wanting either to reject the norm altogether or maintain the status quo.

## 4 Discussion

In India, the slow progress in delaying women's early marriage may reflect the difficulty in shifting socio-cultural norms around marriage and education. However, we have limited understanding of how norms change, and whether they vary by socio-demographic characteristics. Using data from a cohort of parent-adolescent dyads from rural Maharashtra, we investigated whether there were generational changes in social norms, and whether adolescents aspired to shift these norms further for their future hypothetical children, and by how much. We examined whether norms differed by child sex, adolescent sex, or by the adolescent's educational and marital status.

### 4.1 Education

We found that the cohort mothers reflected prevailing education norms in rural Maharashtra, but that the cohort fathers and adolescents of both sexes had substantially surpassed these norms. Education has therefore changed substantially in the more recent generations. This increased education may reflect the parental generation's own desire and ability to change the experience of their children. Where the adolescents specified how much education they would like for their hypothetical children, it was most commonly the same amount that they already had. This indicates that adolescents did not want to shift norms beyond what they themselves had already attained. Importantly, the majority of adolescents were happy for their hypothetical child to decide how much education to complete for themselves, though this might either mean staying in school for longer, or leaving earlier. Less educated adolescents and early-married girls were more likely to want to shift education norms. In contrast, a study from Bihar, India found that early-married girls tended to have lower educational aspirations for themselves, and desired to fulfil traditional roles in their marriage, of being a wife and mother (40).

### 4.2 Marriage

We found that the cohort mothers reflected the prevailing marriage norm in rural Maharashtra, and that the adolescent girls had married later, but not by a large amount (2 years). In contrast, adolescent boys (many of whom had not yet married) had substantially surpassing the prevailing norm of marriage at 21 years. However, we do not know how much of this change was already achieved by the cohort father, as we did not ascertain his marriage age. The majority of adolescents of both sexes did not aspire to reject marriage norms altogether, rather they most commonly wanted to delay marriage for both sons and daughters, moreso for sons than daughters, and with this desire stronger among adolescent girls than boys. Paradoxically, adolescent boys appeared to be breaking out of prevailing marriage norms to a greater extent than girls, despite girls being the primary target of policies to delay marriage. More educated adolescents and unmarried girls aspired for greater changes in marriage norms, potentially because they themselves were already breaking with tradition. However, the early-married and unmarried girls equally aspired to shift marriage norms, suggesting that both those who have broken the norm, and those who had to adhere to it, desired a different future for their own children.

### 4.3 Interaction between norms

Overall, our results suggest that adolescents' aspirations to change education and marriage norms for their hypothetical children were modest, given the substantial increase in their own education compared to their parents. This suggests that their educational aspirations are already being met, though they continue to aspire for later marriage of their offspring. The spousal age-gap that both adolescent girls and boys wanted to maintain for their hypothetical children also reflected adherence to legal and cultural norms, including around gendered societal roles. This marriage age gap has been found in other studies (38, 62), suggesting that the norm is practiced near-universally. Our study provides an example from rural Maharashtra of the underlying norms, but further research is required in other contexts.

Evidence of the extent to which increased education is related to changes in marriage and broader gendered cultural norms and practices is inconsistent. Some studies find that the greater educational attainment of girls has been related to their later marriage (63, 64), and more gender equitable attitudes, at least amongst older adolescent girls and boys (35, 65). In contrast, Singh et al. 2023 found that although girls' increased education in India was associated with a decline in early marriage, the median female age at marriage had increased by only 3 years between 1992 and 2021 (18). Interventions promoting education as the key means to delaying marriage have also had mixed results (16, 66–68). In India, conditional cash transfers successfully helped girls to complete secondary school, but girls in the intervention arm still married earlier than the control group (69). These contrasting findings may be explained by the biased nature of education in India, where gendered norms and power imbalances may be reproduced rather than challenged (70, 71). Girls' secondary education is also leveraged as means to improve marriage prospects rather than to creating other life opportunities such as employment (25, 26, 72, 73), though emerging evidence suggests that the rise in girls' education level means that some inevitably men marry less educated men, who are nevertheless from wealthier households than their natal kin (74).

Overall, marriage norms appear harder to shift than education norms (75), and may actually trump, or drive education norms (64, 76). Dowry, which increases with age and education, is one example of this (77, 78). Studies have estimated that each year's delay in marriage would increase girl's education from 0.2 to 0.5 years (76, 79, 80). However, it is possible that this increased education may eventually delay marriage, but slowly, and across generations. Our findings suggest that educating adolescent boys (who are likely to be the main decision-makers of their children's marriage age) may eventually translate into delaying marriage, but only when they themselves are older and married, and have the opportunity to put this desire into practice for their own children. The extent to which they are able to actually shift these norms will however still depend on elderly parents, who hold the norms of the previous generation. Therefore, there is likely to be a generational time-lag between when increased education actually delays marriage.

### 4.4 Shifting norms

More research is needed on how to shift these norms and their structural drivers beyond education (81). In particular, until the overall role of women in society shifts from that of wife and mothers, substantial delays in female age at marriage and first child bearing are unlikely (58). Dejaeghere et al. explain that although norms may be influenced by more schooling, they are embedded in complex gendered social structures which are slow to change (63). Consistent and even progress is required across the different types of norms: socio-cultural and legal norms, as well as aspirations to shift these. Sustained change in social norms is unlikely to occur unless gender and power structures are addressed at not only the individual, group, and community levels, but also within institutions (81).

For example, a longitudinal study comparing younger (aged 10–14 years) and older (15–19 years) adolescents in Bihar and Uttar Pradesh found several factors influenced greater gender egalitarian views, including older age, greater education, occupational aspirations, adolescent programmes for girls and role models; whereas household wealth, peer groups for boys, social media and discriminatory practices within families were associated with gender inequitable norms (65). A review of the social norms literature suggested that five interacting factors need to be addressed to understand how norms emerge, are maintained and dismantled: correction of misconceptions, structural changes, legal reforms, role models and power dynamics (82). More longitudinal studies are needed to explore how, and at what pace norms change on not only education and marriage, but also different spheres of gender equality (63, 65). We also need to better understand whether shifts in values and aspirations can actually change the decisions that underlie social norms and normative behaviour patterns.

### 4.5 Limitations and strengths

We categorised norm outcomes according to population norms for education and marriage using NFHS data from the rural district of our study (for girls) and rural Maharashtra (for boys). Clearly, changing the definitions of the categories would yield different values and frequencies, but the overall patterns remain broadly similar. Due to ethical concerns, we did not directly ask adolescents when they themselves wanted to marry and could not determine if what they aspired for their hypothetical children matched their aspirations for themselves. The sample size was small for some analyses, including NFHS data. We lacked information on parental and grandparental perspectives, which may have been influential in shaping adolescents' own outcomes as well as their aspirations. We also lacked data on the cohort fathers' marriage ages. There were small biases in our sample for education norms, where adolescents who rejected norms were more like to have dropped out of school and to have been married earlier compared to who provided numerical response for their children's education. Adolescents who did not know their aspirations for their hypothetical children were more likely to have dropped out of school and married early compared to those who gave a response. By the age of 24 years, 91% of adolescent girls and 27% of boys had married, while 58% of girls and 51% of boys had completed their education. These data will therefore change as more adolescents marry and leave school.

Strengths included a relatively big sample (*n* = 659) for the main analysis. We also had data on education and marriage age across two generations, and aspirations that adolescents had for their hypothetical children. To our knowledge, no study has asked adolescents what they aspired for their hypothetical future children, and then coded these aspirations into norm statuses (e.g., reject, shift or maintain) based on the practiced population norm using NFHS data. However, this approach needs to be tested in other contexts.

### 4.6 Conclusion

Despite interventions to increase girls’ secondary education and greater enforcement of minimum marriage age legislation, progress in delaying women's early marriage has been slow. This slow progress may reflect the difficulty in shifting socio-cultural norms, which shape acceptable education levels and marriage age for girls and boys. Using data on 659 parent-adolescent dyads from rural India, we found that marriage norms were harder to shift than education norms. Secular increases in education have yet to translate into substantially delayed marriage for women. There was minimal gender bias in education aspirations, but adolescents wanted daughters to marry earlier than sons. Further research is needed on how aspirations may be shaped by, and break, social norms. However, marital decisions continue to be made by the parental generation, who are likely to conform to their own generation's norms, suggesting a likely time-lag for increased education delaying marriage.

## Data Availability

The datasets presented in this article are not readily available because they cannot be shared publicly due to requirements of the local ethics committee and privacy concerns. Researchers who adhere to the criteria for accessing confidential data, and who understand the expectations of the local ethics committee and study participants, can request data used in this analysis from the Principal Investigator, Professor CS Yajnik. Requests to access the datasets should be directed to csyajnik@gmail.com.
