# Supplementary material for: Generational trends in education and marriage norms in rural India: evidence from the Pune Maternal Nutrition Study
Source: Front Reprod Health. 2025 Jan 20;6:1329806. doi: 10.3389/frph.2024.1329806 (PMC11788393; doi:10.3389/frph.2024.1329806)
Supplement: Supplementary file 1 [file Table1.docx]

**Supplementary Table S1. Sample description, stratified by adolescent girls and boys** (*n*=659)

|  | **Adolescent boys**  (*n*=349) | | **Adolescent girls**  (*n*=310) | | **Difference**  Adolescent boys - girls | |
| --- | --- | --- | --- | --- | --- | --- |
|  | **F** | **%** | **F** | **%** | ***p-*value^1^** | |
| Maternal marriage age (years)  (missing *n*=30) |  |  |  |  | 0.144 | |
| <19 years | 209 | 63 | 201 | 68 |  |  |
| ≥19 years | 125 | 37 | 94 | 32 |  |  |
|  |  |  |  |  |  | |
| Maternal education (years)  (missing *n*=33) |  |  |  |  | 0.071 | |
| None | 71 | 21 | 65 | 22 |  |  |
| Primary (1-8 years) | 158 | 47 | 160 | 55 |  |  |
| Secondary or higher (≥9 years) | 104 | 31 | 68 | 23 |  |  |
|  |  |  |  |  |  | |
| Paternal education (years)  (missing *n*=33) |  |  |  |  | 0.247 | |
| None | 28 | 8 | 31 | 11 |  |  |
| Primary (1-8 years) | 114 | 34 | 113 | 39 |  |  |
| Secondary or higher (≥9 years) | 191 | 57 | 149 | 51 |  |  |
|  |  |  |  |  |  | |
| Caste affiliation (missing *n*=15) |  |  |  |  | 0.154 | |
| Low (tribal, scheduled) | 35 | 10 | 19 | 6 |  |  |
| Mid (artisan, agrarian) | 80 | 24 | 69 | 23 |  |  |
| High (prestige, dominant) | 225 | 66 | 216 | 71 |  |  |
|  |  |  |  |  |  | |
| Socio-economic status (missing *n*=16) |  |  |  |  | 0.088 | |
| Low | 117 | 34 | 80 | 26 |  |  |
| Mid | 113 | 33 | 112 | 37 |  |  |
| High | 209 | 63 | 113 | 37 |  |  |
|  |  |  |  | |  | |
|  | **Adolescent boys**  (*n*=349) | | **Adolescent girls**  (*n*=310) | | **Difference**  Adolescent boys - girls | |
|  | **Mean** | **SD** | **Mean** | **SD** | **Δ (95% CI)** | ***p-*value^2^** |
| Education (years) (missing *n*=130) | 12.0 | 1.6 | 12.0 | 1.4 | -0.1 (-0.3, 0.2) | 0.328 |
| Age at marriage (years)^3^ | 18.4 | - | 18.7 | 1.4 | - | |
|  |  |  |  |  |  | |
|  | **F** | **%** | **F** | **%** | ***p-*value^1^** | |
| Did not complete 10^th^ standard | 47 | 14 | 34 | 11 | 0.329 | |
| Married <19 years | 1 | 1 | 71 | 23 | - | |

*n,* number*.* F, frequency*.* %, percentage. SD, Standard Deviation. NA, not applicable. ^1^Chi-squared test. ^2^Independent samples *t*-test. ^3^Girls *n*=133, boys *n*=1. -, Not applicable.
