# Supplementary material for: Generational trends in education and marriage norms in rural India: evidence from the Pune Maternal Nutrition Study
Source: Front Reprod Health. 2025 Jan 20;6:1329806. doi: 10.3389/frph.2024.1329806 (PMC11788393; doi:10.3389/frph.2024.1329806)
Supplement: Supplementary file 2 [file Table2.docx]

**Supplementary Table S2. Bias, stratified by adolescents who gave numerical response (norm shifters/maintainers) vs did not give numerical response (norm rejectors) for hypothetical children’s education and marriage** (*n*=618)

|  | **Hypothetical children’s education** | | | | | | **Hypothetical children’s marriage** | | | | | |
| --- | --- | --- | --- | --- | --- | --- | --- | --- | --- | --- | --- | --- |
|  | **Adolescents who gave numerical response**  (*n*=189) | | **Adolescents who did not give numerical response**  (*n*=429) | | **Difference**  Numerical – no numerical response | | **Adolescents who gave numerical response**  (*n*=548) | | **Adolescents who did not give numerical response**  (*n*=70) | | **Difference**  Numerical – no numerical response | |
| **Household traits** | | | | | | | | | | | | |
|  | **F** | **%** | **F** | **%** | ***p-*value^1^** | | **F** | **%** | **F** | **%** | ***p-*value^1^** | |
| Maternal marriage age (years) (missing *n*=30) |  |  |  |  | 0.952 | |  |  |  |  | 0.940 | |
| <19 years | 122 | 66 | 270 | 66 |  |  | 347 | 66 | 45 | 66 |  |  |
| ≥19 years | 63 | 34 | 141 | 34 |  |  | 181 | 34 | 23 | 34 |  |  |
|  |  |  |  |  |  | |  |  |  |  |  | |
| Maternal education (years) (missing *n*=33) |  |  |  |  | 0.809 | |  |  |  |  | 0.079 | |
| None | 41 | 22.3 | 82 | 20 |  |  | 116 | 22 | 7 | 10 |  |  |
| Primary (1-8 years) | 32 | 17.4 | 77 | 19 |  |  | 95 | 18 | 14 | 21 |  |  |
| Secondary or higher (≥9 years) | 111 | 60.3 | 243 | 60 |  |  | 308 | 59 | 46 | 69 |  |  |
|  |  |  |  |  |  | |  |  |  |  |  | |
| Paternal education (years) (missing *n*=33) |  |  |  |  | 0.971 | |  |  |  |  | 0.416 | |
| None | 17 | 9 | 39 | 10 |  |  | 52 | 10 | 4 | 6 |  |  |
| Primary (1-8 years) | 65 | 35 | 144 | 36 |  |  | 187 | 36 | 22 | 33 |  |  |
| Secondary or higher (≥9 years) | 102 | 55 | 219 | 55 |  |  | 280 | 54 | 41 | 61 |  |  |
|  |  |  |  |  |  | |  |  |  |  |  | |
| Caste affiliation (missing *n*=15) |  |  |  |  | 0.921 | |  |  |  |  | 0.596 | |
| Low (tribal, scheduled) | 15 | 8 | 37 | 9 |  |  | 48 | 9 | 4 | 6 |  |  |
| Mid (artisan, agrarian) | 42 | 23 | 97 | 23 |  |  | 121 | 23 | 18 | 26 |  |  |
| High (prestige, dominant) | 129 | 69 | 283 | 68 |  |  | 365 | 68 | 47 | 68 |  |  |
|  |  |  |  |  |  | |  |  |  |  |  | |
| Socio-economic Status (missing *n*=16) |  |  |  |  | 0.317 | |  |  |  |  | 0.541 | |
| Low | 62 | 33 | 126 | 30 |  |  | 169 | 32 | 19 | 28 |  |  |
| Mid | 56 | 30 | 152 | 36 |  |  | 186 | 35 | 22 | 32 |  |  |
| High | 69 | 37 | 141 | 34 |  |  | 182 | 34 | 28 | 41 |  |  |
|  |  | |  | |  | |  |  |  |  |  | |
| **Adolescent’s traits, age 19 years** | | | | | | | | | | | | |
|  | **Hypothetical children’s education** | | | | | | **Hypothetical children’s marriage** | | | | | |
|  | **Adolescents who gave numerical response**  (*n*=189) | | **Adolescents who did not give numerical response**  (*n*=429) | | **Difference**  Numerical – no numerical response | | **Adolescents who gave numerical response**  (*n*=548) | | **Adolescents who did not give numerical response**  (*n*=70) | | **Difference**  Numerical – no numerical response | |
|  | **Mean** | **SD** | **Mean** | **SD** | **Δ (95% CI)** | ***p-*value^2^** | **Mean** | **SD** | **Mean** | **SD** | **Δ (95% CI)** | ***p-*value^2^** |
| Education (years) (missing *n*=130) | 12.4 | 1.0 | 12.5 | 0.8 | -0.1 (-0.3, 0.1) | 0.266 | 12.4 | 0.9 | 12.7 | 0.7 | -0.2 (-0.4, -0.1) | **0.025** |
| Age at marriage (years) (missing *n*=525) | 18.5 | 1.5 | 18.9 | 1.4 | -0.4 (-1.0, 0.1) | 0.107 | 18.7 | 1.4 | 19.1 | 1.2 | -0.4 (-1.2, 0.4) | 0.290 |
|  |  |  |  |  |  | |  |  |  |  |  |  |
|  | **F** | **%** | **F** | **%** | **OR (95% CI)** | ***p-*value^1^** | **F** | **%** | **F** | **%** | **OR (95% CI)** | ***p-*value^1^** |
| Did not complete 10^th^ standard | 30 | 16 | 39 | 9 | **1.9 (1.1, 3.1)** | **0.014** | 61 | 11 | 8 | 11 | 1.0 (0.4, 2.1) | 0.941 |
| Married <19 years | 30 | 16 | 32 | 8 | **2.3 (1.4, 4.0)** | **0.001** | 57 | 10 | 5 | 7 | 1.5 (0.6, 13.9) | 0.393 |

*n,* number*.* F, frequency*.* %, percentage. SD, Standard Deviation. OR, Odds Ratio. ^1^Chi-squared test. ^2^Independent samples *t*-test.
