# Supplementary material for: Generational trends in education and marriage norms in rural India: evidence from the Pune Maternal Nutrition Study
Source: Front Reprod Health. 2025 Jan 20;6:1329806. doi: 10.3389/frph.2024.1329806 (PMC11788393; doi:10.3389/frph.2024.1329806)
Supplement: Supplementary file 3 [file Table3.docx]

**Supplementary Table S3. Bias, stratified by adolescents who responded to norm questions vs those who did not respond** (*n*=659)

|  | **Adolescents who responded to norm questions**  (*n*=618) | | **Adolescents who said ‘don’t know’ to norms questions**  (*n*=41) | | **Difference**  Adolescents who responded – those who did not respond | | |
| --- | --- | --- | --- | --- | --- | --- | --- |
| **Household traits** | | | | | | | |
|  | **F** | **%** | **F** | **%** | ***p-*value^1^** | | |
| Maternal marriage age (years) (missing *n*=30) |  |  |  |  | 0.673 | | |
| <19 years | 392 | 66 | 25 | 63 |  |  |  |
| ≥19 years | 204 | 34 | 15 | 38 |  |  |  |
|  |  |  |  |  |  | | |
| Maternal education (years) (missing *n*=33) |  |  |  |  | 0.130 | | |
| None | 123 | 21 | 13 | 33 |  |  |  |
| Primary (1-8 years) | 109 | 19 | 9 | 23 |  |  |  |
| Secondary or higher (≥9 years) | 354 | 60 | 18 | 45 |  |  |  |
|  |  |  |  |  |  | | |
| Paternal education (years) (missing *n*=33) |  |  |  |  | 0.488 | | |
| None | 56 | 10 | 3 | 8 |  |  |  |
| Primary (1-8 years) | 209 | 36 | 18 | 45 |  |  |  |
| Secondary or higher (≥9 years) | 321 | 55 | 19 | 48 |  |  |  |
|  |  |  |  |  |  | | |
| Caste affiliation (missing *n*=15) |  |  |  |  | 0.703 | | |
| Low (tribal, scheduled) | 52 | 9 | 2 | 5 |  |  |  |
| Mid (artisan, agrarian) | 139 | 23 | 10 | 24 |  |  |  |
| High (prestige, dominant) | 412 | 68 | 29 | 71 |  |  |  |
|  |  |  |  |  |  | | |
| Socio-economic Status (missing *n*=16) |  |  |  |  | 0.440 | | |
| Low | 188 | 31 | 9 | 22 |  |  |  |
| Mid | 208 | 34 | 17 | 42 |  |  |  |
| High | 210 | 35 | 15 | 37 |  |  |  |
|  |  | |  | |  | | |
| **Adolescent’s traits, age 19 years** | | | | | | | |
|  | **Adolescents who responded to norm questions**  (*n*=618) | | **Adolescents who said ‘don’t know’ to norms questions**  (*n*=41) | | **Difference**  Adolescents who responded – those who did not respond | | |
|  | **Mean** | **SD** | **Mean** | **SD** | **Δ (95% CI)** | | ***p-*value^2^** |
| Education (years) (missing *n*=130) | 12.4 | 0.9 | 12.2 | 1.3 | 0.3 (-0.2, 0.8) | | 0.252 |
| Age at marriage (years) (missing *n*=525) | 18.7 | 1.4 | 18.7 | 1.4 | 0.1 (-0.7, 0.8) | | 0.929 |
|  |  |  |  |  |  | | |
|  | **F** | **%** | **F** | **%** | **OR (95% CI)** | ***p-*value^1^** | |
| Did not complete 10^th^ standard | 69 | 11 | 12 | 29 | **3.2 (1.6, 6.7)** | **<0.001** | |
| Married <19 years | 62 | 10 | 10 | 24 | **2.9 (1.4, 6.2)** | **0.004** | |

*n,* number*.* F, frequency*.* %, percentage. SD, Standard Deviation. OR, Odds Ratio. ^1^Chi-squared test. ^2^Independent samples *t*-test.
