# Supplementary figures and images for: Generational trends in education and marriage norms in rural India: evidence from the Pune Maternal Nutrition Study
Source: Front Reprod Health. 2025 Jan 20;6:1329806. doi: 10.3389/frph.2024.1329806 (PMC11788393; doi:10.3389/frph.2024.1329806)

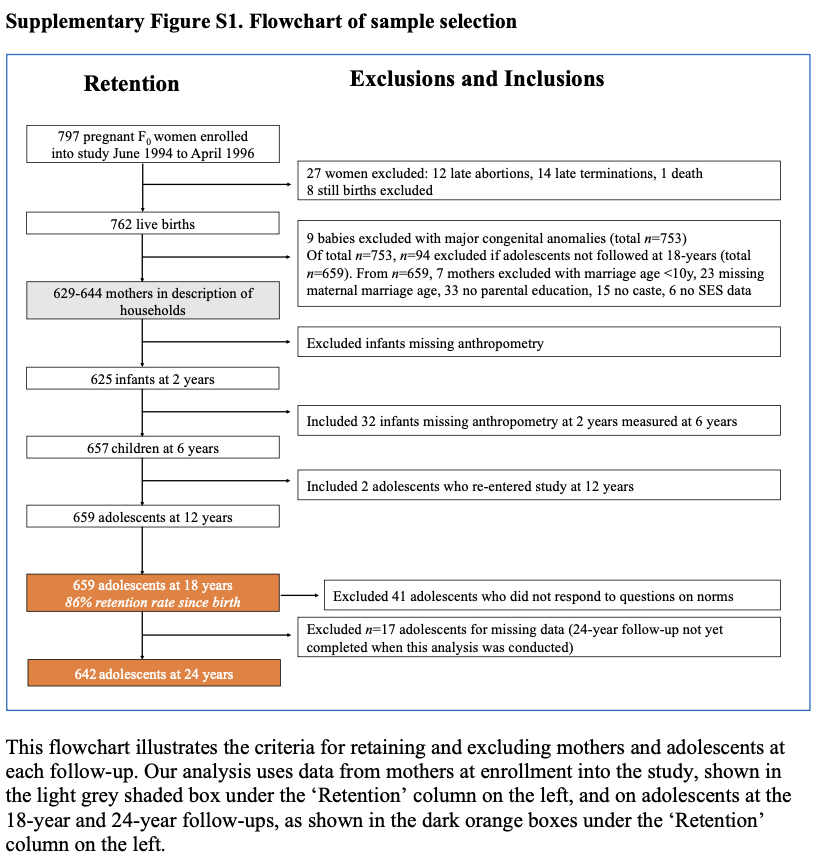

Supplement: Supplementary file 4 [file Image1.tiff]
